# A Multi-User, Single-Authentication Protocol for Smart Grid Architectures

**DOI:** 10.3390/s20061581

**Published:** 2020-03-12

**Authors:** Ahmed S. Alfakeeh, Sarmadullah Khan, Ali Hilal Al-Bayatti

**Affiliations:** 1 Faculty of Computing and Information Technology, King Abdulaziz University, Jeddah 21589, Saudi Arabia; asalfakeeh@kau.edu.sa; 2 School of Computer Science and Informatics, De Montfort University, Leicester LE1 9BH, UK; alihmohd@dmu.ac.uk

**Keywords:** authentication, secret session key, smart grid

## Abstract

In a smart grid system, the utility server collects data from various smart grid devices. These data play an important role in the energy distribution and balancing between the energy providers and energy consumers. However, these data are prone to tampering attacks by an attacker, while traversing from the smart grid devices to the utility servers, which may result in energy disruption or imbalance. Thus, an authentication is mandatory to efficiently authenticate the devices and the utility servers and avoid tampering attacks. To this end, a group authentication algorithm is proposed for preserving demand–response security in a smart grid. The proposed mechanism also provides a fine-grained access control feature where the utility server can only access a limited number of smart grid devices. The initial authentication between the utility server and smart grid device in a group involves a single public key operation, while the subsequent authentications with the same device or other devices in the same group do not need a public key operation. This reduces the overall computation and communication overheads and takes less time to successfully establish a secret session key, which is used to exchange sensitive information over an unsecured wireless channel. The resilience of the proposed algorithm is tested against various attacks using formal and informal security analysis.

## 1. Introduction

Electricity is one of the major stakeholders in the development of the global economy. However, traditional electricity systems fail to reliably react to the current electricity demands due to their one-way communication, centralised generation and electromechanical structure [1]. A new centralised gird technology, the smart grid, has been introduced to overcome the traditional electrical system issues. A smart grid is based on the modern and advanced communication infrastructure that supports a bidirectional communication and energy exchange among the end users (i.e., consumer and providers). For example, a smart meter collects information about electricity usage of the consumer and sends this information to the utility servers. In return, smart meters receive some commands from the utility servers to execute proper actions. In a smart grid, the flow of information is divided into different levels. The first level of information exchange is between the smart devices and smart meters using small-range communication technologies, such as Bluetooth, Zigbee and 6LoWPAN. The second level of information flow is between the smart meter and utility provider and data centres using medium- and long-range communication technologies, such as public telephony networks, the internet and mobile networks [2]. This bidirectional communication plays an important role in balancing the demand and response by controlling power generation based on its utilisation.

It is revealed by the U.S. energy department that, since 1988, there is currently an overall increase of 30% in electricity demand while the peak demand will grow to 20% in the near future. However, there is only a 15% increase in the production and operational efficiency. According to the National Institute of Standards and Technology framework for smart grids, the main tasks are to efficiently handle and process data to ensure better service availability to the end users [3]. Various smart devices are being developed for the demand–response management in smart grids, in which important information is exchanged among the devices and the utility servers for control and operation. This information is prone to numerous cyber attacks, such as replay, impersonation and man-in-the-middle attacks, which can compromise the users’ privacy and modify information [4]. Therefore, communication security is the most critical issue in smart grids, and special attention is needed to protect the channels from these well-known attacks.

A symmetric key-based cryptographic algorithms, like the advance encryption standard, can be used to secure the communication between smart grid and service providers. In these approaches, a unique secret key is shared between each pair of communicating parties. Each secret key is valid only for secure communication between each pair of devices, and it cannot be used for secure communication with other devices of the network. In the last decade, several key management protocols have been proposed to manage those symmetric keys in the context of smart grids. Fouda et al. [5] presented a Diffie–Hellman (DH)-based key establishment protocol, while Zhou [6] presented an elliptic curve based key establishment protocol leveraged on public key infrastructure (PKI) for smart grids in 2011. However, Xia and Wang [7] showed that Zhou’s scheme is vulnerable to the man-in-the-middle attack and needs high maintenance. Furthermore, they proposed a trusted party-based lightweight directory access protocol to reduce the overhead introduced by the PKI. In 2013, Park et al. [8] showed that Xia’s protocol suffers from the unknown key share and impersonation attacks. In 2015, Tsai et al. [9] presented a key distribution protocol that strongly preserves anonymity in the smart grid scenario, but Odelu et al. [10] recently showed that Tsai’s protocol suffers from the ephemeral secret key leakage attack. Finally, He et al. [11] and Mohammadali et al. [12] presented a cost efficient key establishment protocols that have better communication and computational overheads compared with the previous approaches.

A detailed and careful analysis of the existing approaches reveal that three approaches [9,10,11] provide anonymity in smart grid architecture. However, these approaches suffer from the key escrow problem. The main reason for the key escrow problem is the trusted anchor, which is responsible for generating the private key for the smart grid devices and service servers. The key establishment approach, presented in [11], also suffers from private key leakage as is vulnerable to the known session-specific information attacks. These issues motivated us to develop an authentication and key establishment mechanism that can cope with the existing challenges.

### 1.1. Main Contributions

The key contributions of the proposed approach are as follows:

- Developing a lightweight and fast group-based authentication mechanism that executes a full authentication process during the first handshake, while the subsequent handshakes are performed using the authentication token;
- Reducing the energy consumption by reducing the communication overhead; and
- Security validation of the proposed algorithm using a formal and informal security analysis. An Automated Validation of Internet Security Protocol and Applications (AVISPA) tool is used to test the resilience of the proposed algorithm.

### 1.2. Paper Organization

The rest of the paper is organised as follows: A literature survey is provided in Section 2. The preliminaries, including the threat model, solution overview, and system model are discussed in Section 3. Section 4 discusses the proposed group-based authentication and session key establishment mechanism while its formal and informal security validation is discussed in Section 5. The performance evaluation of the proposed approach is presented in Section 6. Finally, Section 7 concludes the paper.

## 2. Literature

Recently, many authentication mechanisms have been proposed for smart grid architectures. Tsai et al. [9] have presented an identity-based key distribution, encryption and signature mechanism for smart grid communication between the smart meter and service provider. Both end devices establish a mutual secret key for the authentication and secure communication. However, Odelu et al. [10] found and proved that this approach is vulnerable to the ephemeral key leakage attacks and the privacy of the smart meters can be compromised. To overcome the aforementioned challenges, Odelu proposed a new authentication mechanism for a smart grid environment.

Doh et al. [13] and Saxena et al. [14] proposed an authentication mechanism among the end users in smart grid architecture that verify their authenticity to prevent any insider and outsider attacks. He et al. [11] presented an elliptic curve cryptography (ECC)-based key distribution mechanism for the smart grid that has less computation cost and lower communication overhead as compared to the Tsai’s scheme [9].

Yan [15] conducted a detailed survey on cyber security in the smart grid. In this survey, IEC 62,351 was presented, which addressed many security features for real-time communication in smart grids. More specifically, it was designed to provide data integrity and authentication using digital signatures and hash functions. An access control mechanism is used to stop unauthorised access to network devices and data while the malicious activities are monitored by the intrusion detection system. Standard hashing algorithms, such as Secure Hash Algorithms (SHA) and Message Digest-5 (MD5), are used to generate a hash from the data and then digitally sign it. The digital signature and its verification are done by the private key and the public key of the sender, respectively. In the verification process, the receiver decrypts the received hash by the sender’s public key and generates a new hash from the received data with the same hashing algorithm used at the sender side. It then compares the decrypted hash with the newly generated hash. The receiver accepts the data if both hashes are equal; otherwise, it discards the data. This verification process is expensive in terms of time and energy consumption, and it is not highly suitable for time-critical applications/processes in the industries.

Tsang [16] presented a fast and secure mechanism for Supervisory Control and Data Acquisition (SCADA) systems. His approach has utilised the concept of a bump in a wire along with the Hash-based Message Authentication Code (HMAC) and Advance Encryption Standard (AES) in the SCADA network. AES is a symmetric key based security algorithm in which each device shares a unique common secret key with every device of the network for secure communication. This approach is suitable for ad hoc and controlled networks as compromising one device can put the whole network security at risk. In a smart grid scenario where each controlling device can be controlled locally and remotely, this approach is highly vulnerable to man-in-the-middle attacks, key compromission attacks, and forgery attacks.

Perrig [17] and Ciarns [18] presented Rivest Shamir Adleman (RSA) based security mechanism for smart grid applications. They utilised the concepts of Message Authentication Code (MAC) and one-time signature (OTS) for data integrity and authentication purposes. Timed Efficient Stream Loss-Tolerant Authentication (TESLA) is a time-slot-based MAC approach where time is divided into slots of fixed duration. For each slot, the sender uses a different key to sign a message. The sender makes the key available to the public as the slot duration expires. Nobody will be able to sign a message with that key for that specific slot as the slot duration has already expired, and the key will be invalid for signing. However, the key will be used to verify the signatures of all the messages signed during that time slot. After the sender makes the key public, it uses another key to sign messages in the next time slot. This approach is not suitable for time-critical applications of the smart grid as it suffers from significant latency in verifying the messages. This is because the first receiver needs to buffer all the received messages before it receives the key. In addition, it is a memory-expensive approach and it is not suitable for resource-constrained devices, especially if the duration of the time slot is large or many messages are generated in each time slot. One time signature approach can solve the issue of replay attacks, but it also suffers from the large computational and communication overhead as HMAC.

## 3. Preliminaries

Here, a brief overview of the adopted threat model and the proposed algorithm is described. Table 1 shows the notations used in this paper.

### 3.1. Threat Model

A well-known threat model, the Dolev Yao (DY) model [19], is considered in this paper to evaluate the performance of the proposed algorithm. In this model, end devices communicate with each other over a public and insecure communication channel, and an adversary *A* has the capabilities to intercept all the messages over this insecure public channel. We are considering the following key features of an adversary:

- **Concurrent execution**—the adversary has the capabilities to start multiple sessions with several devices in parallel so that he/she can act as a man in the middle;
- **Access to public information**—the adversary has full access to all the available public parameters of each device in the network;
- **Message tampering**—the adversary has the capabilities to capture all the messages, tamper them without knowing the actual content and replay them.

The purpose of this threat model is to evaluate the performance of the proposed algorithm and show that an adversary cannot successfully recover information from the captured messages, even if he/she actively participates during the communication. This helps to ensure the security of communication from the man in the middle attacks, replay attacks and forgery attacks.

### 3.2. Algorithm Overview

The proposed algorithm for the authentication and session establishment is divided into five different phases, which are as follows: (1) setup, (2) registration, (3) creation of access policy, (4) authentication mechanism, and (5) session key establishment. During the setup phase, the TTP selects and assigns an elliptic curve to the end devices along with a one-time-secret-token (OTST) and its signed hash. The TTP also defines an access policy for each utility server that (1) limits their access to the smart grid devices and (2) speeds up the authentication process of smart grid devices. During the authentication phase, both the smart grid device and the utility server mutually authenticate each other, followed by session key establishment. A symmetric session key is established to secure messages over insecure public channels. The TTP and utility server use their pre-shared secret key for secure communication with each other. In the proposed algorithm, all mathematical operations are based on the elliptic curve’s mathematics.

### 3.3. System Model

**Figure 1:**
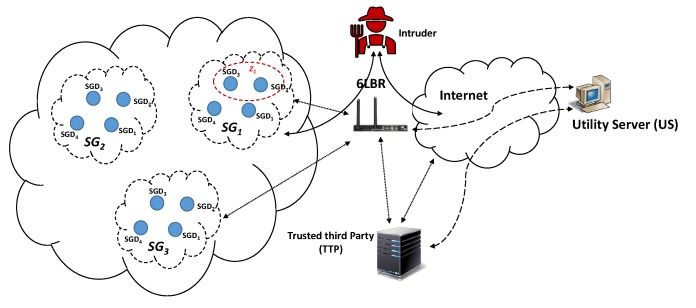
System model.

The proposed system model for smart grid consists of utility server (US) and smart grid devices (SGDs; i.e., smart meter and other smart devices). As shown in Figure 1, smart grid devices are used for controlling and monitoring various factors and plays an important role in the demand–response management by communicating with the utility server. These devices are grouped together to form a group, $SG_{i}$. The devices within a group (i.e., $SGD_{1}$, $SGD_{2}$, $SGD_{3}$, …) can further be grouped together into various small groups (i.e., known as $Z_{i}$) of different sizes such that $Z_{1}\cap Z_{2}=⌀$. These devices are connected to the internet through a border gateway router (6LBR). The TTP registers devices and utility servers by validating their OTST and signs certificate for their public keys using its own private key. It also defines an access policy for the utility servers. Each utility server accesses these devices based on pre-defined access policy. For example, it can easily be imagined by considering a large building automation process where different areas are leased to different companies. Each company is given some access rights to control various appliances (i.e., lights, heating, ventilation, etc) in its leased own area and not in other parts of the building. Depending on the employee’s role, the company can also restrict employee’s access to a specific unit(s), for example, devices belonging to $Z_{i}$.

In this paper, a multicast communication architecture is considered where the utility server (US) establishes a communication link with multiple smart grid devices ($SGD_{i}$). For each communication session, US and $SGD_{i}$ authenticate each other.

### 3.4. Elliptic Curve Description and Notations

An elliptic curve (EC) $E_{p}\left(a,b\right):y^{2}=x^{3}+ax+b$ (mod *p*) is defined over a finite field *p*, where *p* is a large prime number. The domain parameters of the elliptic curve are represented by (p,a,b,G,n), where *n* is a large prime number and *G* is a base point generator. Each end device (US or SGD) generates its public key (*K*) and private key (PK) using the EC Diffie–Hellman (ECDH) approach, where *K* = PK×G. These devices receive a certificate (Cert) for their public keys from the TTP. The Cert issuance is discussed in the following section.

## 4. Proposed Algorithm

In this section, various steps of the proposed algorithm are described in detail.

### 4.1. Setup

During the setup phase, the network devices are configured as follows:

***Step-1:***  Each device of the network is equipped with a non-singular EC $E_{P}\left(a,b\right):y^{2}=x^{3}+ax+b$ (mod *p*) over a finite field $Z_{P}$, where *a* and *b* are constants and satisfy the condition $4a^{3}+27b^{2}\neq 0$ and $Z_{P}=\left\{0,1,2,\ldots ,P-1\right\}$;
***Step-2:***  A device is given a non collision hash function H(.);
***Step-3:***  A device selects *G* (group generator). The order of *G* is set to *n* and satisfies the condition n.G=℘, where *℘* represents the point at infinity or zero; and
***Step-4:***  The TTP selects a private key $PK_{TTP}\in Z_{P}$ on elliptic curve and calculates its private key $K_{TTP}=PK_{TTP}\cdot G$; and
***Step-5:***  The TTP makes $K_{TTP}$, H(.) and $E_{P}\left(a,b\right)$ public.

### 4.2. Registration

During the registration phase, SGD and US register themselves with the TTP and get all the necessary secret information from the TTP which are utilised during the authentication and key establishment phases.

#### 4.2.1. Smart Grid Device Registration

- The TTP randomly selects a unique identity ($ID_{SGD}$) for the smart grid device and calculates $sgID=H(PK_{TTP}||ID_{SGD})$;
- The TTP calculates a timestamps $TS_{SGD}=H(PK_{TTP}\left|\right|T_{reg})$, where $T_{reg}$ represents the registration time of the SGD with the TTP. This $TS_{SGD}$ is used to generate a new timestamps for the SGD communication with the US and TTP after the registration phase;
- The TTP generates a $OTST=H(PK_{TTP}||T_{reg||ID_{SGD}})$; and
- The TTP gives $\langle sgID,TS_{SGD},OTST\rangle$ offline to the SGD.
- The TTP pre-load the usIDj onto the SGD memory, where j={1,2,3,…}.

#### 4.2.2. Utility Server Registration

- The TTP randomly selects a unique identity ($ID_{US}$) for the utility server and calculates $usID=H(PK_{TTP}||ID_{US})$;
- The TTP calculates a timestamps $TS_{US}=H(PK_{TTP}\left|\right|T_{reg})$, where $T_{reg}$ represents the registration time of the US with the TTP. This $TS_{US}$ is used to generate a new timestamps for the US communication with the SGD and TTP after the registration phase;
- The TTP generates a $OTST=H(PK_{TTP}||T_{reg||ID_{US}})$;
- The TTP gives $\langle usID,TS_{US},OTST\rangle$ offline to the US; and
- The TTP pre-load the sgIDi onto the US memory where i={1,2,3,…}.

#### 4.2.3. Cert Generation

The SGD uses its certificate to incorporate the certificate mechanism for the verification of its public key, while the public key is embedded in the access policy ID (apID). After the deployment of SGD, it requests a certificate from the TTP. To this aim, the following steps are executed by the SGD and US:

- The SGD selects a random number $r_{SGD}\in \left[1,n-1\right]$ and current timestamps *T*;
- The SGD calculates $R_{SGD}=r_{GD}\times G$ and a verification hash $VH_{SGD}=H(TS_{SGD}\left||T||OTST\right)⊕H(sgID||R_{SGD}\left||T\right)$;
- The SGD sends $\langle R_{SGD},VH_{SGD},T,H\left(OTST\right)\rangle$ to the TTP;
- The TTP verifies the received H(OTST) by comparing it with $H(H(PK_{TTP}\left|\right|T_{reg}||ID_{SGD}))$;
- The successful verification of OTST and $VH_{SGD}$ allows the TTP to generate a certificate $Cert_{SGD}=H\left(R_{SGD}+r_{TTP}\times G\right)=H\left(R_{SGD}+R_{TTP}\right)$ and a signature $s=H(PK_{TTP}+r_{TTP})$; and
- Finally, the TTP sends $\langle Cert_{SGD},s,VH_{TTP},T\rangle$, where $VH_{TTP}=H(Cert_{SGD}\left||T||OTST\right)⊕H(s||TS_{SGD}\left||T\right)$.

The US also requests TTP in a similar way for its certificate as described above for the SGD.

#### 4.2.4. Public/Private Key Generation

After receiving the $Cert_{SGD}$, the SGD generates a private and public keys pair as follows:

(1) $$PK_{SGD}=s+r_{SGD}\times H(Cert_{SGD}\left||sgID\right)$$

(2) $$K_{SGD}=PK_{SGD}\times G$$

Similarly, the US generates its private and public key pair as

(3) $$PK_{US}=s+r_{US}\times H(Cert_{US}\left||usID\right)$$

(4) $$K_{US}=PK_{US}\times G$$

The utility server (US) can compute the public key of SGD using the $Cert_{SGD}$ and the public key ($K_{TTP}$) of the TTP as

(5) $$K_{SGD}=K_{TTP}+Cert_{SGD}\times H(Cert_{SGD}\left||sgID\right)$$

The utility server (US) receives $Cert_{US}$ for its public key $K_{US}$ from the TTP and embeds it along with its $K_{US}$ in apID. The US uses the apID for the authentication of its public key ($K_{US}$) to the SGD. The pubic keys are used for the ECDH key exchange. Both the SGD and the US compute authentication key AK after exchanging their Cert and *K* with each other as follows:

(6) $$AK=PK_{SGD/US}\times K_{US/SGD}$$

### 4.3. Access Policy

**Figure 2:**
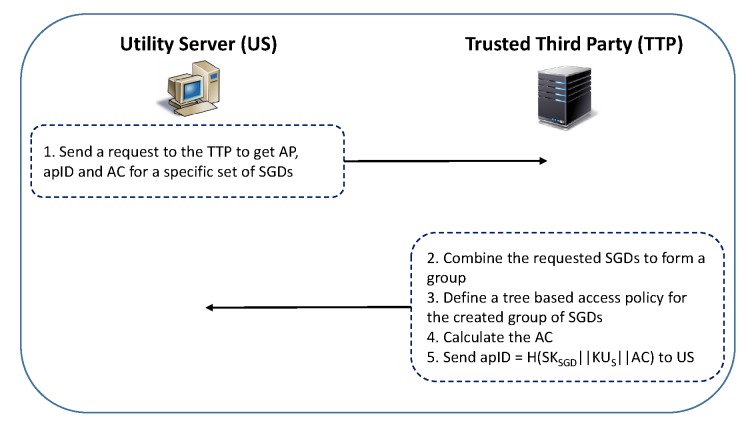
A utility server request to the trusted third party to get access policy, its ID, and authentication code.

To access smart grid devices (SGDs), after generating its public/private key pair, each US requests the TTP to issue an access policy (AP) along with its ID (apID) and an authentication code (AC). Figure 2 shows the pictorial representation of the said request–response messages exchanged between the US and TTP.

**Figure 3:**
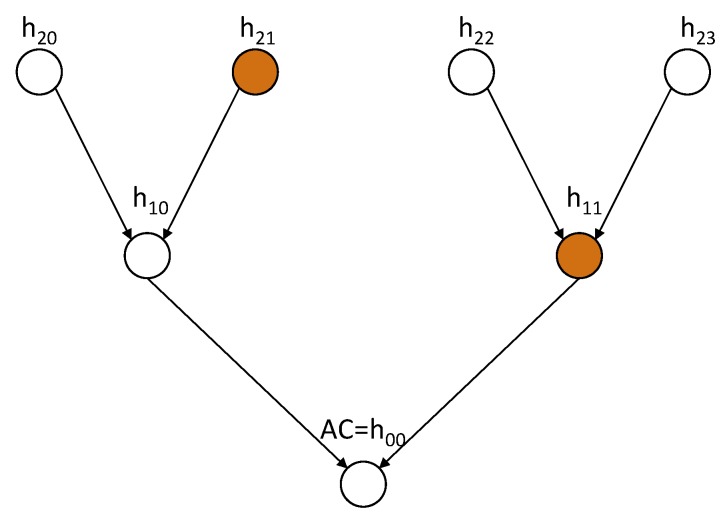
Access policy for $SG_{1}$ = {$SGD_{1},SGD_{2},SGD_{3},SGD_{4}$}.

This request contains utility server ID (US), list of the SGDs and utility server’s public key ($K_{US}$). For example, US wants access to $SGD_{1},SGD_{2},SGD_{3}$ and $SGD_{4}$. The TTP follows the following steps to generate AP and AC:

- All intended SGDs are grouped together to form a group (i.e., SG as shown in Figure 1) such that $i=2^{l}$, where *i* is total number of SGDs in a smart grid and l≥1;
- The access policy is defined as a tree structure, where the leaf nodes are the hashes of SGDs and the root hash is the authentication code (AC), as shown in Figure 3;
- The hashes of $SGD_{1},SGD_{2},SGD_{3},SGD_{4}$ are represented by $h_{(log_{2}i)0},h_{(log_{2}i)1},\ldots ,h_{\left(log_{2}i\right)\left(i-1\right)}$; and
- The authentication code is $AC=h_{00}=H(h_{10}\left|\right|h_{11})$, where $h_{jk}=H(h_{\left(j+1)(2k\right)}\left|\right|h_{\left(j+1)(2k+1\right)})$ for $j=0,1,\ldots ,(log_{2}i)-1$ and k=0,1,2,…,i-1.

It is clear from Figure 3 that $h_{10}$ and $h_{11}$ are the child hashes of the root hash ($h_{00}$, considered as AC) and the leaf hashes are the child hashes of $h_{10}$ and $h_{11}$. Therefore, AC can be easily calculated using the leaf hashes and subsidiary hashes. For example, AC can be calculated from $h_{21}$ and $h_{11}$ if $h_{20}$ is given. This is because, $h_{10}=h_{20}\left|\right|h_{21}$ and $AC=h_{00}=h_{10}\left|\right|h_{11}$ This approach generates the AC more quickly and consumes fewer computational resources.

Once the AP is defined and constructed by the TTP, $apID=H(SK_{SGD}||K_{US}||AC)$ is issued by the TTP to the utility server, where $SK_{SGD}$ is the symmetric key that TTP pre-shares with the SGD. This response includes apID, the set of SGDs (e.g., $SG_{1}$), and the public key of the TTP ($apID,SG_{1},K_{TTP}$). Algorithm 1 shows the summary of all steps involved in obtaining the apID and AC values.
**Algorithm 1** Generation and issuance of access policy, apID and authentication code by the KMS to the utility server1:$TTP\leftarrow utilityserver:US,SGD,K_{US}$2:Construction of AP3:$AC\leftarrow H_{00}=H(h_{10}\left|\right|h_{11})$4:$TTP\leftarrow US:US,SGD,K_{US}$5:$apID\leftarrow H(SK_{SGD}||K_{US}||AC)$6:$utilityserver\leftarrow TTP:apID,SG_{1},K_{TTP}$

### 4.4. Authentication and Session Key Establishment

The proposed authentication approach consists of the two following phases: (1) initial handshake and (2) subsequent handshake. The initial handshake mechanism uses the certificate and public/private key pair to authenticate end devices and to generate AK. This AK serves as the pre-shared secret key (PSK) and helps in calculating the TK, which is generated at the end of the initial handshake. The subsequent handshakes use the AK for authentication and verification purposes. The utility server uses AK for the authentication of other SGDs within the same group (e.g., $SG_{1}$). Thus, US does not need a public key after the initial handshake.

During the authentication phase, the following steps are employed:

- The US selects a random number $w\in Z_{P}$ and generates a current time stamp $T_{1}$;
- The US calculates a nonce $N_{US}=w\cdot G$, $XB=H(TS_{US}||T_{1})$ and $TS_{1}=H(TS_{US}\left|\right|T_{1})⊕H(sgID||N_{US}\left|\right|T_{1})$. The sgID is pre-shared with the utility server by the TTP during the registration phase;
- The US sends $\langle N_{US},TS_{1},XB,T1\rangle$ to the SGD;
- The SGD validates the timestamp $T_{1}$ after receiving $\langle N_{US},TS_{1},T1\rangle$ by checking $|T_{1}-T_{1}^{*}|\leq △T$, where △T is the maximum propagation time of a message over a channel. It also checks the validity of $TS_{1}$;
- If $T_{1}$ and $TS_{1}$ are valid, the SGD selects a random number $v\in Z_{P}$ and generates a current time stamp $T_{2}$;
- The SGD calculates a nonce $N_{SGD}=v\cdot G$ and $TS_{2}=H(TS_{SGD}\left|\right|T_{2})⊕H(sgID||N_{SGD}\left|\right|T_{2})⊕H(Cert_{SGD}\left|\right|T_{2})$;
- The SGD calculates $TX=v\cdot N_{us}=vwG$, $B=H(sgID||N_{US}||T_{2})$ and $XT=TS_{1}⊕H(sgID||N_{US}\left|\right|T_{1})=H(TS_{US}\left|\right|T_{1})$;
- The SGD calculates the corresponding SSK using the key-generation function (KF) as SSK=KF(AK,H(TX||XT||B));
- The SGD further calculates $Q=H(SSK||usID||T_{2})$ and $R=B⊕H(usID||N_{SGD}||N_{us}||T_{2})$;
- The SGD sends $\langle N_{SGD},TS_{2},Cert_{SGD},Q,R,T_{2}\rangle$ to the US;
- The US validates the timestamp $T_{2}$ after receiving $\langle N_{SGD},TS_{2},Cert_{SGD},Q,R,T_{2}\rangle$ by checking $|T_{2}-T_{2}^{*}|\leq △T$, where △T is the maximum propagation time of a message over a channel;
- The successful validation of $T_{2}$ allows the US to compute the public key of $SGD_{1}$ using Equation (5);
- The US then computes the AK using Equation (6), $AK=PK_{US}\times K_{SGD}=PK_{US}PK_{SGD}G$;
- To calculate the SSK, the US first computes $E=R⊕H(usID||N_{SGD}||N_{us}||T_{2})$ and $TX^{*}=w\cdot N_{SGD}$;
- The US then computes $SSK^{*}=KF(AK,H(TX^{*}||E||H(TS_{us}\left|\right|T_{1})))$;
- The successful verification of *R* and *Q* allows the US to send the apID and subsidiaries of SGD to SGD and a hash of $SSK^{*}$ (i.e., $H(SSK^{*})$);
- The SGD then computes AK using Equation (6) and verifies the received hash ($H(SSK^{*})$) by comparing it with its computed hash from the SSK;
- Successful verification of the received hash allows SGD to compute the AC, deriving the apID as $H(SK_{SGD}||K_{US}||AC)$ and comparing it with the received apID. Successful verification allows the utility server to establish a secure link with $SGD_{1}$; and
- Finally, $SGD_{1}$ calculates the TK=[US,AK,AC]CK1 and sends it to the utility server where CK1 is a common key shared among the $SG_{1}$ devices.

The utility server uses the TK along with the AK with any SGD in $SG_{1}$ in the subsequent handshakes for authentication. If the utility server wants to authenticate another SGD of the same $SG_{1}$, they first exchange the nonce with each other. The utility server then shares the TK with $SGD_{2}$. $SGD_{2}$ computes the AK using Equation (6) and AC from the AP. Then, it obtains the AC from the received TK as it has the CK1 and compares it with the calculated AC. Successful verification allows the utility server to access $SGD_{2}$ and both derive SSK. Finally, both verify the hash of the SSK.

## 5. Security Analysis

In this section, we show the capabilities of the proposed algorithm to resist some of the well-known attacks by providing formal and informal security analysis.

### 5.1. Formal Security Analysis Using AVISPA

We validate the reliability of the proposed algorithm using AVISPA tool [20,21]. AVISPA is a standard tool use to analyse the security strength of the security algorithm. A security algorithm is modelled within the AVISPA tool using a human readable High Level Protocol Specification Language (HLPSL), which is then automatically translated into intermediate Format (IF) for formal security analysis by using four attack models (SAT-based Model-Checker (SATMC), TA4SP, On-the-Fly Model-Checker (OFMC) and CL-based Model-Checker (CL-AtSe)).

**Figure 4:**
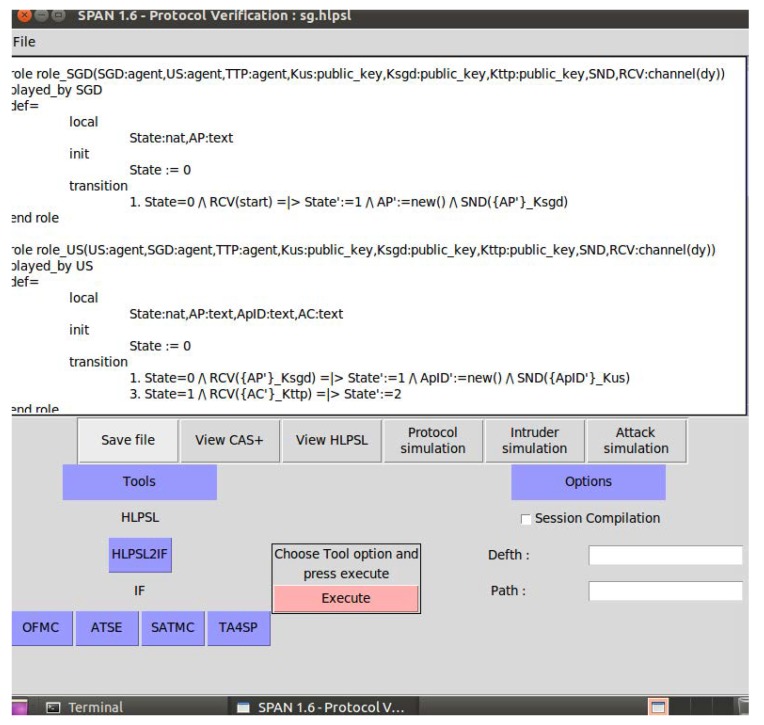
Screen shot of the simulation tool.

Figure 4 shows the screen shot of algorithm testing using the AVISPA-SPAN tool. The proposed algorithm is implemented as follows:

1. First, all the public and private parameters and the communication links among the smart grid devices are defined;
2. All the messages are scheduled in sequence and properly labelled. For example, US sends a message to SGD. It is labelled as 1 while the response from the SGD is labelled as 2. The next message from the US is labelled as 3 and so on;
3. The total number of messages per device and the content of each message are defined;
4. Then, the role and capabilities of an attacker are defined as man-in-the-middle where it has full access to all the messages being exchanged among the devices in the smart grid;
5. Finally, all the security sensitive parameters are defined; and
6. The AVISPA attack models are run to check the security strength of the proposed algorithm.

**Figure 5:**
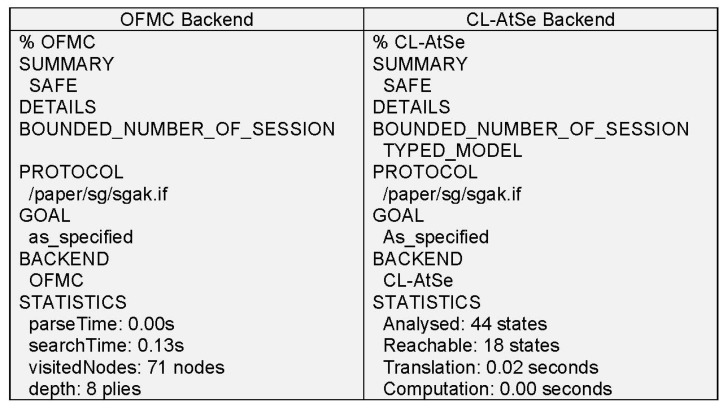
Output of the SPAN-AVISPA analysis.

Figure 5 represents the outcomes of the AVISPA test and the strength of the proposed algorithm.

### 5.2. Informal Security Analysis

In this section, we informally analyse the proposed algorithm to show that its effectiveness against the following attacks.

#### 5.2.1. Man-In-The-Middle Attack (MMA)

A man-in-the-middle attack is implemented by introducing a fake device between the US and SGD. Such attacks are difficult to detect because the attacker impersonates an authentic SGD/US. This attack allows the attacker to easily manipulate the captured packets or send fake data if the implemented algorithm is not secure against this attack. In the proposed algorithm, this attack is possible during the certificate extraction phase and during the authentication and session key establishment phase.

During the certificate generation phase as described in Section 4.2, the SGD sends $\langle R_{SGD},VH_{SGD},T,H\left(OTST\right)\rangle$ to the TTP. If the attacker in the middles changes $R_{SGD}$ to $R_{SGD}^{*}$, the attacker will not be able to change $VH_{SGD}=H(TS_{SGD}\left||T||OTST\right)⊕H(sgID||R_{SGD}\left||T\right)$, which is dependent on $R_{SGD}$. This is because the attacker does not have any information about the $TS_{SGD}$ and OTST and while the TTP knows the values of $TS_{SGD}$ and OTST. If the TTP receives a modified message $\langle R_{SGD}^{*},VH_{SGD},T,H\left(OTST\right)\rangle$ from the attacker, it would not be able to verify $VH_{SGD}$ with the new $R_{SGD}^{*}$. The unsuccessful verification will alert the TTP about the possible man-in-the-middle attack. Similarly, the SGD receives a certificate from the TTP as $\langle Cert_{SGD},s,VH_{TTP},T\rangle$. If the attacker changes the certificate to $Cert_{SGD}^{*}$, it will not be able to change the $VH_{TTP}=H(Cert_{SGD}\left||T||OTST\right)⊕H(s||TS_{SGD}\left||T\right)$. This is because the attacker does not know the exact values of actual $R_{SGD}$, OTST and $TS_{SGD}$. If the SGD device receives a modified message $\langle Cert_{SGD}^{*},s*,VH_{TTP},T\rangle$ from the attacker, it will not be able to verify $VH_{TTP}$ which will alert the SGD about the possible man-in-the-middle attack.

During the authentication phase, the US sends $\langle N_{US},TS_{1},XB,T\rangle$ to the SGD. If the attacker changes the nonce to $N_{US}^{*}$, the attacker will not be able to change the $TS_{1}=H(TS_{US}\left|\right|T_{1})⊕H(sgID||N_{US}\left|\right|T_{1})$ as these changes need $TS_{US}$ and sgID which are not known to the attacker. Thus, upon receiving $\langle N_{US}^{*},TS_{1},XB,T\rangle$ by the SGD, it will not be able to verify the $TS_{1}$. This is because $TS_{1}⊕XB=H(sgID||N_{US}\left|\right|T_{1})\neq H(sgID||N_{US}^{*}\left|\right|T_{1})$. This will alert the SGD about the possible man-in-the-middle attack. Similarly, the SGD sends $\langle N_{SGD},TS_{2},Cert_{SGD},Q,R,T_{2}\rangle$ to the US after receiving and verification of the authentication request from the US. If the attacker changes $N_{SGD}$ to $N_{SGD}^{*}$ and $Cert_{SGD}$ to $Cert_{SGD}^{*}$, the US will receive a modified message $\langle N_{SGD}^{*},TS_{2},Cert_{SGD}^{*},Q,R,T_{2}\rangle$. The US will verify *Q* and *R* which are dependent on $N_{SGD}$, $N_{US}$, SSK and usID. Only the authentic SGD knows these values and the attacker does not have any information about them. To verify *R*, the US validates $E=H(sgID||N_{US}||T_{2})$. To do so, $E=R⊕H(usID||N_{SGD}^{*}\left|\right|N_{US}\left|\right|T_{2})\neq H(sgID||N_{US}\left|\right|T_{2})$ because $N_{SGD}\neq N_{SGD}^{*}$. This will alert the US about the possible man-in-the-middle attack and it will result in the wrong SSK generation and invalidate the value of *Q*, which is dependent on SSK. Thus, if the attacker makes some changes within the message, it is immediately detected by the end devices. The reason is that the attacker only knows the public information of both US and SGD and does not have any information about their private information.

In the case of the public/private key encryption/decryption mechanism, the attacker can only decrypt a message using the sender’s public key if and only if it is encrypted with the sender’s private key. After decryption, it is not possible for the attacker to re-encrypt the message using the sender’s private key as the attacker does not know the sender’s private key. If the message (i.e., secret information) is encrypted with the public key of the receiver, then the attacker is not able to decrypt it as it does not know the private key of the receiver. In conclusion, the proposed algorithm is secure against the man-in-the-middle attacks.

#### 5.2.2. Replay Attacks

**Figure 6:**
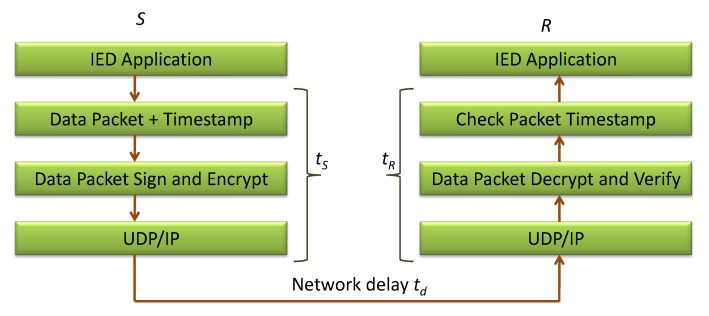
Time consumed in various stages.

Sometimes, the attacker does not modify the content of the messages, but it delays or replays the messages to disturb the normal functionality of the smart grid. In the proposed approach, we use the timestamps in the messages as shown in the general representation of communication between the end devices in Figure 6. During the registration phase, the SGD sends $\langle R_{SGD},VH_{SGD},T,H\left(OTST\right)\rangle$ to the TTP which includes the timestamp *T*. The TTP checks $T-T^{*}\leq △T$, where $△T=t_{max}$ represents the maximum delay that a message can encounter during its transmission between the end devices. If the time check condition is valid, the message is accepted as a new and fresh message. The attacker cannot modify the timestamp in the message as *T* is also involved in calculating $VH_{SGD}$. Similarly, the TTP sends $\langle Cert_{SGD},s,VH_{TTP},T\rangle$ to the SGD which also includes *T* to verify the freshness of the message.

During the authentication phase, the US sends $\langle N_{US},TS_{1},T1\rangle$ to the SGD and the SGD sends $\langle N_{SGD},TS_{2},Cert_{SGD},Q,R,T_{2}\rangle$ to the US which also includes $T_{1}$ and $T_{2}$ to verify the freshness of the messages, where $TS_{1}$ and $TS_{2}$ are dependent on $T_{1}$ and $T_{2}$, respectively.

These timestamps play an important role in verifying the packet creation time and its validity. If the timestamp is not expired (i.e., less than $△T=t_{max}$), the receiving device will accept the packet; otherwise, it will reject the packet. In this way, the proposed security approach works well against the replay attacks.

#### 5.2.3. Forgery of the apID

As described above, the utility server can access only a limited number of SGDs that are defined by its access policy (e.g., [$SGD_{1},SGD_{2},SGD_{3},SGD_{4}$] or [$SGD_{2},SGD_{3}$]). The generated SSK can only be used with a device with which it is in communication. This key cannot be used with any other device in the same group or in a subset of a group. This is because it is dependent on each individual $N_{SGD}$, $N_{US}$, sgID, $TS_{1}$, and $TS_{2}$, which are unique for each individual SGD and US. The forgery of the utility server’s apID means that an adversary (adv) can compute the ECDH public/private key pair (i.e., K,PK) such that

(7) $$H(SK_{SGD}\left|\right|K_{US}||AC)=H(SK_{SGD}\left|\right|K_{adv}+\left||AC\right)$$

where $apID=H(SK_{SGD}||K_{US}||AC)$ is the actual utility server’s apID. Given the $apID=H(SK_{SGD}||K_{US}||AC)$, it not computationally feasible to find $apID^{\prime }=H(SK_{SGD}\left|\right|K_{adv}\left||AC\right)$. This is because H(.) is the preimage resistance hash function, where the output can easily be calculated using the inputs, but it is very difficult to calculate or predict the input values based on the given output. Therefore, it is not possible to find this private key by knowing only the public key.

Similarly, as described above, the forgery attack is also not possible during the certificate generation phase and authentication and session key establishment phase. This is because the attacker does not know the private parameter assigned to the US and SGD, which play an important role in authenticating and verifying the messages exchange during the certificate generation and authentication process.

#### 5.2.4. End-to-End Security

If both the utility server and SGD have already shared their public keys with each other, then there is no need for the certificate. This is because, during the first handshake/session establishment, both end users use their certificate to verify their public keys to each other. In the subsequent sessions, they do not need to verify their public keys again. However, this is not always true. In most cases, SGDs are replaced or upgraded with new SGDs or SGD changes the US, and they need to re-authenticate themselves with the utility servers, even if they belong to the same group or subgroup. Therefore, there is always a need for a certificate to verify and authenticate a public key of the end user. Thus, the certificate plays an important role in maintaining the end-to-end security.

## 6. Simulation Setup and Results

The performance of the proposed approach is evaluated using MATLAB and OMNeT++. MATLAB is used to simulate the power system [22], while OMNeT++ is used to evaluate the communication performance. The UDP is used instead of TCP to avoid any retransmission delay and 100-Mbps links are used for connections. An adaptive scheduler is used to integrate MATLAB and OMNeT++ because all operations of smart grids depend on the actions and decisions of the controllers, while the scheduler synchronises the operation of both simulators.

### 6.1. Communication Cost

To calculate the communication cost during the authentication phase, we consider the length of random number (i.e., nonce), certificate and apID as 160, 320 and 832 bits, respectively. The selection of 160 bits for ECC-based calculations was made according to the NIST recommendations, which state that the security of a 160-bit EEC-based system is equivalent to a 2048-bit RSA system [23]. During the initial handshake for authentication, US and SGD exchange three messages in total (i.e., request (nonce), cert and apID) requiring 160 bits, 320 bits and 832 bits and results in 1312 bits communication overhead. The subsequent authentication with the same SGD or other SGDs with the same group needs to exchange AK and TK with a valid time-stamp requiring 160 bits, 160 bits and 32 bits, respectively. This results in 352-bit communication overhead during the subsequent handshakes. Table 2 shows the comparison of proposed algorithm with other schemes. The schemes of Fouda [5], Wu and Zhou [6], Xia [7], Tsai-Lo [9], Odelu et al. [10], and He [11] require 6804, 4248, 2768, 3520, 3840 and 1760 bits, respectively. It is clear that our scheme requires minimum communication cost compared with those schemes.

### 6.2. Cost of Cryptographic Operations

Time is one of the critical factors in smart grid communication, playing an important role in the system’s stability and synchronisation. The performance of the proposed algorithm in terms of time consumption during the cryptographic operations are observed using a MICAZ device (as SGD) and with a 3-GHz Pentium IV system as a utility server. The processing time of various cryptographic operations is shown in Table 3.

The overall computational costs of the proposed algorithm and various existing algorithms are shown in Table 4. The following notations are used to represent various computational costs:

- $T_{KF}$: computational time of key generation function;
- $T_{PM}$: computational time of point multiplication;
- $T_{E_{S}}$: computational time of symmetric encryption;
- $T_{E_{P}}$: computational time of public key encryption;
- $T_{H}$: computational time of hash function;
- $T_{sig}$: computational time of signature;
- $T_{ver}$: computational time of signature verification; and
- $T_{add}$: computational time of ECC point addition.

Based on the computational cost presented in Table 4 and the cost of each cryptographic operation shown in Table 3, the comparative analysis of the total time consumed by the proposed algorithm during the authentication and session key with other existing schemes is shown in Table 5.

The cryptographic operations in the proposed algorithm are based on elliptic curve mathematics, and the key length is fixed to 160-bits according to the NIST recommendations. Each cryptographic mechanism needs a key specific length to achieve a certain level of security. For example, for a security level set by the NIST that an RSA-based approach can achieve with a key of 2048 bits in length, the same security can be achieved by a 160-bit key in the elliptic curve-based approach, which is much shorter than the RSA based approach. Similarly, a 256-bit key is needed for DSA. Using short keys improves the performance of security algorithms as they consume less time during the verification and encryption process.

### 6.3. Memory Cost

Table 6 shows the total memory occupied by the secret keys use in the authentication and secure channel establishment process. It is clearly evident from the results that the proposed mechanism needs fewer keys for both authentication and secure channel establishment and occupies less memory if compared with [24] and RSA mechanism. Although there is little difference in the memory requirements if compared with [25] and DSA, but there is a big difference in the end-to-end delay as discussed in the previous section. The memory requirement of the TV-HORS mechanism is extremely high as it needs more than 500 KB of memory to store secret keys for the authentication and secure channel establishment.

### 6.4. Security Comparison

Table 7 shows the security comparison of the proposed algorithm against some of the existing algorithms in terms of the man in the middle attack, impersonation attack, forward secrecy, replay attacks, mutual authentication feature, formal and informal security validation. It is clear from the comparative analysis that the proposed algorithm successfully addresses all the described features, and it is suitable for the smart grid applications.

## 7. Conclusions

This paper presented a policy-based group authentication approach for smart grid architectures where multiple SGDs are authenticated by the utility server in a fast, reliable, and secure manner. Section 4 described the proposed algorithm step-by-step and explained the importance/utilization of all the important parameters during the registration phase, authentication and key establishment phases. It also described how to use the access policy during the authenticate process and how to limit the access of a utility server to the smart grid devices. The formal and information security validation of the proposed algorithm is carried out with a well-known security validation tool AVISPA and with a mathematical proof, respectively. Both the security validation approaches verified the soundness of the proposed algorithm against well known replay attacks, man-in-the-middle attack and forgery attack. The performance evaluation of the proposed algorithm in Section 6 showed that it generates less communication overhead, consumes less memory to store the secret parameters and consumes less computation resources, which makes it a better choice for the resource constrained devices of a smart grid. This is because the first authentication requires all the steps to be followed, while the subsequent authentications with the same and other SGDs are done using the authentication token. This approach has made the proposed mechanism lightweight due to fewer computational requirements and made it more robust against forgery, man-in-the-middle and replay attacks. The comparison of the security features of the proposed algorithm with the state of the art showed that the proposed algorithm is robust to more attacks than the state-of-the-art approaches.

In the future, we will try to further optimise and enhance the standard security protocols for smart grid communication, where an internet utility server is used to access any internet connected devices and has the ability to control different functionality of the smart grid devices.

## Figures and Tables

**Table 1:** Notation table.

Parameters	Definition
$t_{d}$	Message delivery time
$t_{S}$	Time spent in cryptographic operations at the sender side
$t_{R}$	Time spent in the verification operation at the receiver side
$d_{msg}$	Size of message in bits
$t_{max}$	Maximum acceptable communication delay
*N*	Nonce
US,SGD	Utility server and smart grid device
TTP	Trusted third party
*K*, PK	EC public key and EC private key
SK	Symmetric key shared between TTP and SGD
Cert	Public key certificate
KF(.)	Key generation function
H(.)	Second preimage resistant hash function
AP, apID	Access policy and access policy ID
AC, AK	Authentication code and authentication key
SSK	Session secret key
TK	Token key
CK	Common key among all devices of the SG

**Table 2:** Comparison of communication costs.

Algorithm	Cost (bits)	No. of Messages
[5]	6804	4
[6]	4248	6
[7]	2768	5
[9]	3520	3
[10]	3840	3
[11]	1760	3
Ours (initial)	1312	3
Ours (subsequent)	352	2

**Table 3:** Computational time of various cryptographic operations.

Operation	MICAZ	3-GHz Pentium IV PC
Key generation	5.32 s	3.88 ms
Point multiplication	2.45 s	1.82 ms
AES en/decryption	0.023 ms	∼0 ms
Hash function	0.023 ms	∼0 ms
Public encryption	0.79 s	0.57 ms
Public decryption	21.5 s	16 ms
Signature	21.5s	16 ms
Signature verification	0.79 s	0.57 ms
ECC point addition	0.44 ms	∼0 ms

**Table 4:** Computational operations of various algorithms.

Algorithm	Cryptographic Operations
[6]	$8T_{PM}+1T_{E_{S}}+1T_{sig}+1T_{ver}+5T_{H}$
[9]	$7T_{PM}+2T_{add}+2T_{KF}+2T_{E_{P}}+10T_{H}$
[10]	$5T_{PM}+2T_{add}+2T_{KF}+2T_{E_{P}}+12T_{H}$
[11]	$10T_{PM}+3T_{add}+11T_{H}$
Proposed (initial)	$4T_{PM}+3T_{H}+3T_{KF}+1T_{ver}$
Proposed (subsequent)	$2T_{PM}+1T_{KF}+1T_{ver}$

**Table 5:** Computational times of various algorithms.

Algorithm	MICAZ (s)	Pentium IV PC (ms)
[6]	41.91	31.1
[9]	29.37	21.6
[10]	24.47	18.0
[11]	24.50	18.2
Proposed (initial)	26.55	19.5
Proposed (subsequent)	11.10	8.1

**Table 6:** Memory occupied by the security parameters.

Algorithm	Security Parameters (bits)
[5]	3392
[6]	3232
[7]	2208
[9]	3072
[10]	5120
[11]	1632
Proposed	960

**Table 7:** Security comparison of various algorithms.

Algorithm	S1	S2	S3	S4	S5	S6	S7
[5]	*√*	*√*	*√*	*√*	*√*	*√*	χ
[6]	χ	*√*	χ	*√*	χ	χ	χ
[7]	*√*	χ	χ	*√*	χ	χ	χ
[9]	*√*	*√*	*√*	*√*	*√*	*√*	χ
[10]	*√*	*√*	*√*	*√*	*√*	*√*	χ
[11]	*√*	*√*	*√*	*√*	*√*	*√*	χ
[12]	*√*	*√*	*√*	*√*	*√*	χ	*√*
Ours	*√*	*√*	*√*	*√*	*√*	*√*	*√*

Note: S1.MMA resistance. S2. Resistance against Impersonation attack. S3. Forward secrecy feature. S4. Resistance against replay attack. S5. Mutual authentication. S6. Informal security validation. S7. AVISPA security validation.
